# Method of computing direction-dependent margins for the development of consensus contouring guidelines

**DOI:** 10.1186/s13014-021-01799-1

**Published:** 2021-04-13

**Authors:** Liam S. P. Lawrence, Lee C. L. Chin, Rachel W. Chan, Timothy K. Nguyen, Arjun Sahgal, Chia-Lin Tseng, Angus Z. Lau

**Affiliations:** 1 Department of Medical Biophysics, University of Toronto, Toronto, ON Canada; 2 Department of Medical Physics, Odette Cancer Centre, Toronto, ON Canada; 3 Department of Radiation Oncology, Sunnybrook Health Sciences Centre, University of Toronto, Toronto, ON Canada; 4 Physical Sciences Platform, Sunnybrook Research Institute, 2075 Bayview Ave., Toronto, M4N 3M5 ON Canada; 5 Department of Radiation Oncology, London Health Sciences Centre, Western University, London, ON Canada

**Keywords:** Consensus contouring, Clinical target volume margins, Interobserver variability, Stereotactic body radiotherapy

## Abstract

**Background:**

Clinical target volume (CTV) contouring guidelines are frequently developed through studies in which experts contour the CTV for a representative set of cases for a given treatment site and the consensus CTVs are analyzed to generate margin recommendations. Measures of interobserver variability are used to quantify agreement between experts. In cases where an isotropic margin is not appropriate, however, there is no standard method to compute margins in specified directions that represent possible routes of tumor spread. Moreover, interobserver variability metrics are often measures of volume overlap that do not account for the dependence of disagreement on direction. To aid in the development of consensus contouring guidelines, this study demonstrates a novel method of quantifying CTV margins and interobserver variability in clinician-specified directions.

**Methods:**

The proposed algorithm was applied to 11 cases of non-spine bone metastases to compute the consensus CTV margin in each direction of intraosseous and extraosseous disease. The median over all cases for each route of spread yielded the recommended margins. The disagreement between experts on the CTV margin was quantified by computing the median of the coefficients of variation for intraosseous and extraosseous margins.

**Results:**

The recommended intraosseous and extraosseous margins were 7.0 mm and 8.0 mm, respectively. The median coefficient of variation quantifying the margin disagreement between experts was 0.59 and 0.48 for intraosseous and extraosseous disease.

**Conclusions:**

The proposed algorithm permits the generation of margin recommendations in relation to adjacent anatomy and quantifies interobserver variability in specified directions. This method can be applied to future consensus CTV contouring studies.

**Supplementary Information:**

The online version contains supplementary material available at 10.1186/s13014-021-01799-1.

## Background

Conformal radiotherapy techniques, including stereotactic radiosurgery (SRS) and stereotactic body radiotherapy (SBRT), are increasingly being delivered for most malignancies. Consistent and accurate delineation of the clinical target volume (CTV) is essential for ensuring treatment efficacy and meaningful interpretation of dosimetric and clinical outcomes in clinical trials. The CTV is defined as an additional margin to the gross tumor volume (GTV), the demonstrable extent of the tumor, to encompass microscopic disease. Ideally, CTV margin recommendations are informed by pathological evaluation of microscopic tumor extension [1–3] and examination of patterns of disease recurrence. In the absence of such data, expert consensus can provide guidance. Over the last decade, a number of consensus contouring studies to formulate guidelines have emerged for various disease sites [4–12].

In these studies, experts are typically provided with patient images and asked to contour the CTV given a reference GTV. Measures of inter-observer variability are used to determine the agreement between experts [13]. A consensus CTV is often created from a collection of expert contours by applying the simultaneous truth and performance level estimation (STAPLE) algorithm [14]. Consensus contouring recommendations typically include CTV margins based, in part, on the STAPLE consensus CTV. In clinical contouring practice, the GTV would be expanded isotropically or anisotropically along the Cartesian axes using these margins.

However, the extent of tumor spread can vary in arbitrary directions because of anatomic barriers, anisotropic tissue structure, or preferential migration within the tissue of origin [15]. For certain tumor sites, CTV contouring guidelines that account for this complexity may be more appropriate than isotropic margin recommendations. In spine SRS, for example, the CTV is defined by vertebral sectors depending on GTV involvement instead of by a simple isotropic margin [5]. Automatic CTV delineation using vertebral sectors has already been implemented in treatment planning systems [16]. Future treatment planning software should also facilitate expansion in arbitrary directions using anisotropic margins for better conformation to the disease volume.

For making contouring recommendations by direction, a method of analyzing expert contours to generate margins in specified directions that represent potential routes of tumor spread is needed. Such analysis would also benefit from a metric for quantifying interobserver variability in specified directions to assess disagreement on the appropriate margin. Common measures of interobserver variability like the Dice coefficient and the kappa statistic [17] are based on volume overlap and therefore do not capture the dependence of disagreement on direction. Existing algorithms that could be applied for computation of anisotropic margins and contour variability rely on approximately spherical volumes [18, 19], which may not be a valid assumption, or do not make use of specified directions [20–22].

This study proposes a novel method to compute consensus CTV margins and margin variability in directions chosen based on clinical assessment of potential routes of spread and anatomical barriers. For generating clinical contouring recommendations, the consensus CTV margin is computed for specified routes of spread in multiple cases, then a margin recommendation is made based on a summary statistic across all cases. For demonstration, the method is applied to 11 cases of non-spine bone metastases, where margins are expected to be anisotropic, to determine separate intraosseous and extraosseous margins.

## Methods and materials

### Metrics

**Fig. 1:**
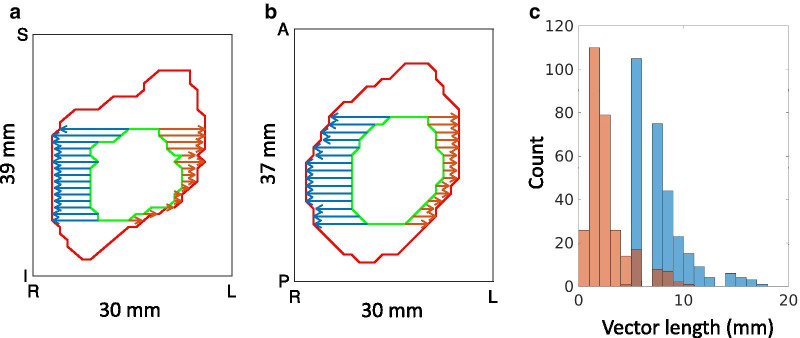
Diagram for directional margin explanation: **a** and **b** show coronal and axial views of the GTV (green) and CTV (red). In blue and orange are the expansion vectors in the anatomical right (R) and left (L) directions respectively. The dimensions are shown along the axes. **c** The vector lengths collected over the surface of the GTV. Computing the medians yields an expansion of 7.0 mm in the R direction and 2.0 mm in the L direction

The upcoming metric definitions rely on a quantity called the *directional margin* $M({\varvec{d}})$, where ${\varvec{d}}$ is any clinically relevant direction in 3D*.* The directional margin is computed from a GTV and a CTV in two steps. First, a set of $N$ *expansion vectors* $\{{{\varvec{v}}}_{i}{\}}_{i=1}^{N}$ are identified: these vectors are oriented parallel to ${\varvec{d}}$ and extend from the GTV surface to the CTV surface without intersecting the interior of the GTV. Second, the directional margin is defined as the median of the lengths of the expansion vectors: $M({\varvec{d}})={\text{MEDIAN}}\left({\left\{\left|{{\varvec{v}}}_{i}\right|\right\}}_{i=1}^{N}\right)$, where $|\cdot |$ is the Euclidean norm. This procedure is illustrated in Fig. 1. The diagram shows 2D contours for demonstration, but the algorithm can be applied to any contiguous 3D volume. Pseudocode is shown in an additional file (see Additional file 1).

The consensus CTV margin is the directional margin of the GTV to the consensus CTV. The margin deviation is the coefficient of variation of the directional margins from the GTV to each expert CTV. For $K$ expert CTVs and a set of directional margins $\{M({\varvec{d}}{)}_{j}{\}}_{j=1}^{K}$ in direction ${\varvec{d}}$, ${\text{CV}}(\mathbf{d})={\text{STD}}\left(\{M({\varvec{d}}{)}_{j}{\}}_{j=1}^{K}\right)/{\text{MEAN}}\left(\{M({\varvec{d}}{)}_{j}{\}}_{j=1}^{K}\right)$ is the margin deviation.

### Application to clinical cases

The method was applied to 11 clinical cases, chosen specifically to represent a wide range of sites, and taken from a study on international practice patterns for the use of SBRT to treat non-spine bone metastases [23]. Each of nine radiation oncologists delineated the CTV contour, based on the provided GTV contour and the simulation CT and MRI scans. The contours were defined in the CT space (in-plane pixel size: 1.17 × 1.17 mm; slice thickness: 1–3 mm). Using all CTV contours, a consensus contour was computed using the STAPLE algorithm and approved by all radiation oncologists for use in making contouring recommendations [23].

For each case, directions of intraosseous and extraosseous extension were identified by two co-authors ([TKN] and [CLT]), both radiation oncologists. In each direction, the consensus margin was computed using the GTV and the consensus CTV, while the margin deviation was computed using the GTV and the individual participant CTVs. Computations were done using MATLAB (version R2016b, The Mathworks, Inc.). The median consensus margin and margin deviation for intraosseous and extraosseous extension were computed across all cases.

## Results

**Fig. 2:**
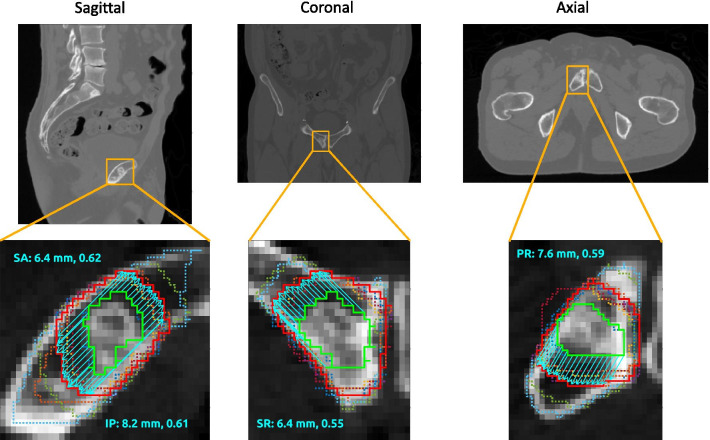
Consensus margin and margin deviation for a metastasis in the pubic symphysis without an extraosseous component: CT and overlaid contours for a patient with a metastasis in the pubic symphysis. The GTV and STAPLE CTV are represented by the solid green and red contours, respectively; the expert contours are dotted. The directions of potential intraosseous extension, indicated by the cyan vectors, are termed *superior-anterior* (SA), *inferior-posterior* (IP), *superior-right* (SR), and *posterior-right* (PR). The text annotations report the consensus margin (mm) followed by the margin deviation (dimensionless) for each direction

**Fig. 3:**
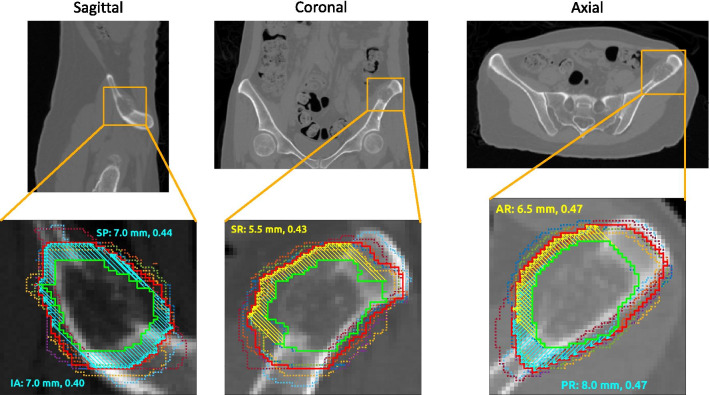
Consensus margin and margin deviation for a metastasis in the iliac crest showing cortical disruption: CT and overlaid contours for a patient with a metastasis in the iliac crest. The GTV and STAPLE CTV are represented by the solid green and red contours, respectively; the expert contours are dotted. The directions of potential intraosseous and extraosseous extension are indicated by the cyan and yellow vectors, respectively. These directions are termed *superior-posterior* (SP), *inferior-anterior* (IA), *superior-right* (SR), *anterior-right* (AR), and *posterior-right* (PR). The text annotations report the consensus margin (mm) followed by the margin deviation (dimensionless) for each direction

Figures 2 and 3 illustrate the results. Figure 2 shows four directions of potential intraosseous extension for a metastasis in the pubic symphysis (the tumor showed no extraosseous component). The directions are termed *superior-anterior* (SA), *inferior-posterior* (IP), *superior-right* (SR), and *posterior-right* (PR). The consensus margins for the four directions were 6.4 (SA), 8.2 (IP), 6.4 (SR), and 7.6 (PR) mm. Figure 3 shows a metastasis in the iliac crest demonstrating cortical disruption. Three directions of potential intraosseous extension were identified: *superior-posterior* (SP), *inferior-anterior* (IA), and *posterior-right* (PR). Two directions of extraosseous extension were also selected: *superior-right* (SR) and *anterior-right* (AR). The intraosseous margins were 7.0 (SP), 7.0 (IA), and 8.0 (PR) mm, while the extraosseous margins were 5.5 (SR) and 6.5 (AR) mm. Note that computing a directional margin allowed separate estimates of intraosseous and extraosseous extension from the same case. The margin deviations for the pubic symphysis case (Fig. 2) were 0.62 (SA), 0.61 (IP), 0.55 (SR), and 0.59 (PR). For the iliac crest case (Fig. 3), the margin deviations for the intraosseous directions were 0.44 (SP), 0.40 (IA), and 0.47 (PR); for the extraosseous directions, the deviations were 0.43 (SR) and 0.47 (AR). The spread of margin deviation values over the different directions for both cases indicates that contouring disagreement can vary by direction.

**Fig. 4:**
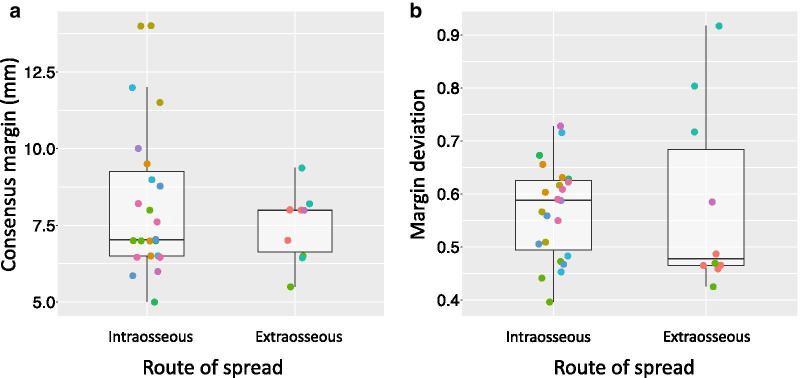
Comparison of intraosseous and extraosseous consensus margins and margin deviations: **a** and **b** show boxplots of the consensus CTV margins and margin deviations over all 11 cases of non-spine bone metastases. The points of a given color are the metrics in each direction for a specific case

Figure 4 shows the consensus margins and margin deviations over all 11 cases. The median intraosseous consensus margin was 7.0 mm (range 5–14 mm). The median extraosseous consensus margin was 8.0 mm (range 5.5–9.4 mm). The median margin deviations were 0.59 (range 0.40–0.73) for intraosseous disease and 0.48 (range 0.43–0.92) for extraosseous disease. These consensus margins can inform contouring recommendations for stereotactic body radiotherapy of non-spine bone metastases.

## Discussion

The purpose of the directional margin algorithm is to quantify CTV expansions by direction for the development of contouring guidelines. The advantage of applying this algorithm over computing an isotropic margin is that the extent of tumor spread may depend on direction because of anatomical barriers or the nature of surrounding tissue. In an application to multiple cases of non-spine bone metastases the proposed method yielded separate estimates of intraosseous and extraosseous extension, which would not have been possible by computing an isotropic margin, as illustrated by the case in Fig. 3. This data can inform the development of subsequent consensus contouring recommendations. Since routes of spread will typically vary in different directions, anisotropic CTV expansions in arbitrary directions should be a feature of future treatment planning systems.

The agreement between experts on the appropriate margin for a given route of spread can be quantified using the margin deviation. The margin deviation was found to vary by direction (Figs. 2 and 3), illustrating the advantage of a direction-dependent measure of interobserver variability. The range of the margin deviation will need to be characterized in more subjects so that outliers can be identified in future studies on contouring variability that employ this technique.

**Fig. 5:**
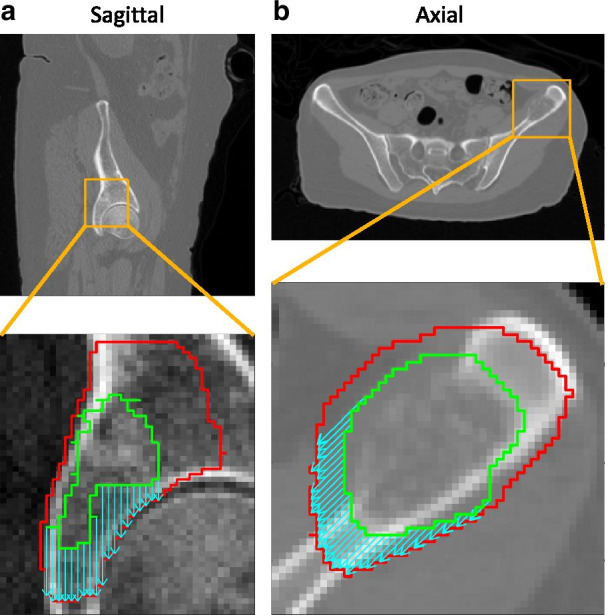
Cases for discussion of strengths and limitations of method: **a** The GTV (green) and STAPLE CTV (red) for an acetabular metastasis. The cyan vectors in the inferior direction, indicating a potential route of intraosseous tumor spread, show variation in length, which is accounted for by the proposed algorithm. **b** The GTV (green) and STAPLE CTV (red) for a metastasis in the iliac crest (same as Fig. 3), with the cyan vectors indicating a potential direction of intraosseous extension. Some of the vectors lie outside of the bone

One of the strengths of the proposed method is the ability to account for complex geometries, since the lengths of all expansion vectors are incorporated (Fig. 5a). The primary limitation of this method is that it does not account for the fact that only a subset of the GTV surface is relevant for margin computation when barriers to tumor spread are present in the specified direction (Fig. 5b). The method can be generalized by segmenting anatomical barriers and excluding inappropriate vectors. This strategy could be facilitated by recent advances in automated segmentation for CTV delineation (15, 24).

Future work will include the application of the presented methods to the same 11 cases to inform contouring recommendations for SBRT of non-spine bone metastases. Applying the method to other tumor sites is another direction for future studies.

## Conclusions

This report described and demonstrated a novel method to compute CTV margins and margin disagreement between expert contours in any number of specified clinically relevant directions. The proposed approach allows for the generation of margin recommendations in relation to adjacent anatomy. The method was applied to 11 cases of non-spine bone metastases to compute recommended intraosseous and extraosseous margins. The margin disagreement was also quantified. The method can be applied in future consensus contouring studies.

## Supplementary Information

**Additional file 1.** Title: Directional margin algorithm pseudocode. Description: Pseudocode for the directional margin algorithm, which forms the basis of the proposed method.

## Data Availability

The datasets used and/or analyzed during the current study are available from the authors on reasonable request.
