# Supplementary material for: Method of computing direction-dependent margins for the development of consensus contouring guidelines
Source: Radiat Oncol. 2021 Apr 13;16:71. doi: 10.1186/s13014-021-01799-1 (PMC8045331; doi:10.1186/s13014-021-01799-1)
Supplement: Supplementary file 1 — Additional file 1. Title: Directional margin algorithm pseudocode. Description: Pseudocode for the directional margin algorithm, which forms the basis of the proposed method. [file 13014_2021_1799_MOESM1_ESM.pdf]

```
DATA: GTV and CTV as 3D logical arrays of the same dimension, a unit vector direction.
RESULT: GTV-to-CTV margin in direction
initialize vector lengths as empty list
FOR each grid point
        IF grid point is inside GTV
                set vector length to 0
                set continue search to True
                WHILE continue search is True
                        add 0.5 to vector length
                        set position to grid point + (vector length x direction)
                        IF position is inside GTV
                                set continue search to False
                                set save line segment to False
                        ELSE IF position is outside CTV
                                set continue search to False
                                set save line segment to True
                        END IF
                END WHILE
                IF save line segment is True
                        subtract 0.5 from vector length
                        append vector length to vector lengths
                END IF
        END IF
END FOR
RETURN median of vector lengths
```

Directional margin algorithm pseudocode: The GTV and CTV are represented by 3D logical arrays. The array indices are considered to be an integer-valued grid in 3D space, where each grid point corresponds to one CT voxel. A position in this 3D space is inside a volume if and only if all 8 surrounding grid points are true. For simplicity, this pseudocode omits a conditional that ensures line segments that overlap with the GTV surface, but not the interior, are excluded.
